# Impact of Pregnancy on Breast Cancer Features and Prognosis

**DOI:** 10.3390/curroncol31040171

**Published:** 2024-04-19

**Authors:** Valentina E. Bounous, Carola Minella, Luca Fuso, Silvia Actis, Greta Petroni, Luca G. Sgrò, Martina Borghese, Nicoletta Tomasi Cont, Riccardo Ponzone, Annamaria Ferrero

**Affiliations:** 1 Gynecology and Obstetrics Unit, Umberto I Hospital, Department of Surgical Sciences, University of Turin, Largo Turati 62, 10128 Turin, Italy; carola.minella@unito.it (C.M.); lfuso@mauriziano.it (L.F.); silvia.actis@unito.it (S.A.); greta.petroni@unito.it (G.P.); lsgro@mauriziano.it (L.G.S.); annamaria.ferrero@unito.it (A.F.); 2 Department of Gynecology and Obstetrics, Santa Croce and Carle Hospital, 12100 Cuneo, Italy; borghese.m@ospedale.cuneo.it; 3 Azienda Sanitaria Locale TO3, 10093 Collegno, Italy; nicoletta.tomasicont@aslto3.piemonte.it; 4 Gynaecological Department, Candiolo Cancer Institute, FPO-IRCCS, Strada Provinciale 142, 10060 Candiolo, Italy; riccardo.ponzone@ircc.it

**Keywords:** breast cancer, pregnancy, pregnancy associated breast cancer (PABC), prognosis

## Abstract

Background: pregnancy-associated breast cancer (PABC) affects one in 3000 pregnancies, often presenting with aggressive features. Methods: We retrospectively evaluated a cohort of 282 young BC patients (≤45 years old) treated between 1995 and 2019, dividing them into three groups: nulliparous women, women with PABC (diagnosed within 2 years since last pregnancy) and women with BC diagnosed > 2 years since last pregnancy. This last group was further stratified according to the time between pregnancy and BC. The analysis encompassed histological factors (tumor size, histotype, grading, nodal involvement, multifocality, lympho-vascular invasion, hormone receptor expression, Ki-67 index, and HER2 expression), type of surgery and recurrence. Results: Age at diagnosis was younger in nulliparous than in parous women (*p* < 0.001). No significant differences were noticed regarding histological characteristics and recurrences. At univariate analysis, nodal involvement (OR = 2.4; *p* < 0.0001), high tumor grade (OR = 2.6; *p* = 0.01), and lympho-vascular invasion (OR = 2.3; *p* < 0.05), but not pregnancy (OR = 0.8; *p* = 0.30), influenced DFS negatively. Multivariate analysis confirmed nodal involvement as the only negative independent prognostic factor for a worse DFS (OR = 2.4; *p* = 0.0001). Conclusions: in our experience, pregnancy is not an independent adverse prognostic factor for BC DFS.

## 1. Introduction

Breast cancer is the most frequently diagnosed cancer in women globally. It, along with cervical cancer, hematological diseases, and melanoma, is also the most common malignancy diagnosed during pregnancy or post-partum [1]. Pregnancy-associated breast cancer (PABC) affects one in every 3000 pregnancies, with an increased incidence in recent years mainly due to higher maternal age at delivery [2].

Definitions of PABC are highly variable in the literature [3,4]. PABC can be referred to as BC diagnosed during pregnancy and within the first year post-partum [5], but also up to 2 or even 5 years post-partum, depending on the study [6,7,8].

Some authors suggest that distinguishing between breast cancer diagnosed during pregnancy and postpartum/lactation could improve the characterization of disease biology and prognosis [9]. In fact, PABC and post-partum BC might present different clinical characteristics [10], and data on prognosis are still highly controversial [11,12]. Lastly, one study focused on differences among PABC diagnosed in different trimesters of gestation, and found better clinicopathological characteristics and prognosis in first-trimester PABC [13].

Pregnancy seems to have a dual effect on BC development—both protective and promotive. Immediately after pregnancy, the risk of BC development increases and then decreases over the years while moving away from pregnancy. Biological features also differ, with BC being more aggressive when diagnosed during or immediately after pregnancy, and then becoming less aggressive, when compared with BC in nulliparous women of the same age [14]. A 25% reduction in the risk of developing the luminal BC subtype was observed in parous women compared with nulliparous ones through the literature [15].

PABC is rare, but carries aggressive features: young age at diagnosis, advanced local stage, negative receptor status or Human Epidermal Growth Factor Receptor 2 (HER 2) overexpression, lympho-vascular invasion (LVSI), and lymph node involvement [16].

In addition, the treatment of PABC can be limited or delayed due to obstetric factors [17]. Breast cancer during pregnancy can be treated with chemotherapy during the second and third trimesters and post-partum. However, radiotherapy, hormone therapy, or biological agents such as trastuzumab are currently not recommended during pregnancy. Surgery is always an option for treatment [18].

Thus, even if there is not complete agreement in the literature [19], PABC usually presents with a worse prognosis [8,20,21].

This study aims to evaluate the impact of pregnancy on the characteristics and prognosis of BC in terms of disease-free survival (DFS) in young patients, considering different groups based on parity and time since the last pregnancy.

## 2. Materials and Methods

This study included 282 young women diagnosed with BC who underwent surgery from 1995 to 2019 at Mauriziano Hospital in Turin and IRCCS in Candiolo (TO). Due to the absence of a universally accepted definition for “young woman”, women aged 45 years or younger were considered. All patients’ information regarding pregnancy and BC features were collected from non-open-access institutional databases.

For this study, were considered “PABC” all BC diagnosed during pregnancy or within two years since pregnancy, according to the studies of Johansson [6] and Gooch [22]. BC patients were divided into three subgroups: nulliparous women, women diagnosed with PABC, and women with BC diagnosed beyond two years after the last pregnancy. Further stratification of the latter group was performed by dividing patients with BC diagnosis between 2 and 5 years after pregnancy, between 5 and 10 years, and beyond 10 years since last pregnancy.

Regarding BC features, tumors were considered hormone receptor-positive if at least 1% of the cells tested expressed estrogen (ER) and/or progesterone receptors (PgR) [23,24]. HER2 overexpression was defined with immunohistochemistry (IHC) staining 3+ or, in the case of equivocal results (2+), with an amplification in situ hybridization (FISH) test [25].

We classified molecular subtypes using IHC surrogates for ER, PgR, HER-2 status, and Ki-67 index, as follows: luminal A (ER and/or PgR positive and HER-2 negative; ki67 < 20%), luminal B (ER and/or PgR positive, HER-2 positive and ki67 > 20%), HER-2 (ER and PgR negative and HER-2 positive), and basal-like (triple negative BC, TNBC) [26,27].

All of the patients who underwent surgery at our institutions were treated in compliance with international guidelines. As for adjuvant or neoadjuvant therapies, all the patients received medical and/or radiotherapeutic treatment according to the prevailing standards of care at the time. It is worth noting that trastuzumab was introduced as an adjuvant therapy for HER2-positive breast cancer patients in our institutions in 2006.

Statistical analysis was performed with SPSS. For analysis of continuous variables, analysis of variance (ANOVA) and post hoc tests were used to evaluate statistically significant differences, with Bonferroni correction for multiple comparisons. To assess the frequency of qualitative variables within the various categories of patients, contingency tables were created, and the chi-square statistical test was used. Survival analysis was performed using Kaplan–Meier curves, subjected to the Log-Rank test. Local and distant recurrence were analyzed separately. Finally, a univariate and a multivariate analysis were performed using Cox’s time-dependent logistic regression model, thus correcting the Odds Ratios for confounding factors. The threshold value of *p* was considered < 0.05.

## 3. Results

**Figure 1:**
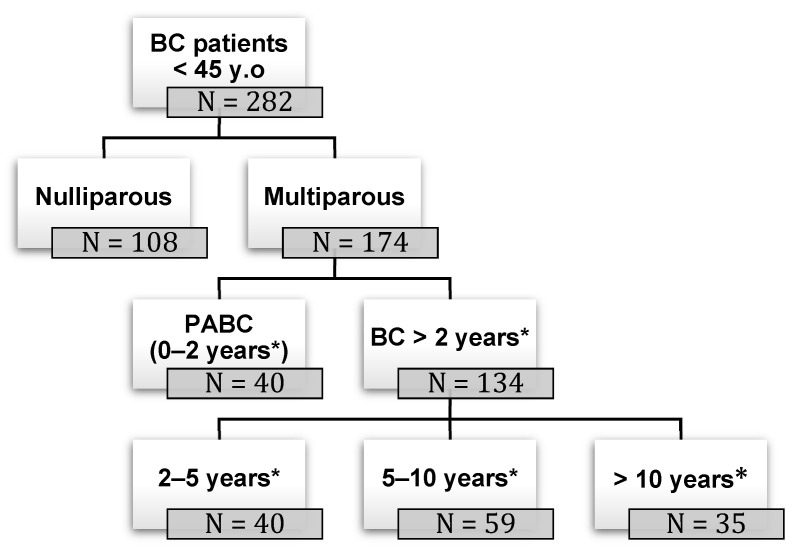
Study population. BC: breast cancer; PABC: pregnancy-associated breast cancer; (*) years since pregnancy at the time of BC diagnosis.

The study analyzed 282 cases of BC in women younger than 45 years old (mean age 36 years old; median age 37 years old). Of these, 108 were nulliparous and 174 were multiparous. Among the multiparous women, 40 patients were diagnosed with BC during pregnancy or within the next two years (PABC), whereas the other 134 cases were diagnosed with cancer subsequently (40 women were diagnosed with BC between 2 and 5 years since their last pregnancy, 59 between 5 and 10 years, and 35 more than 10 years since last pregnancy), as shown in Figure 1.

### 3.1. PABC

The mean age at diagnosis in this group of patients was 36 years old (median age of 37 years old). In 5% of cases (two patients), the diagnosis occurred during pregnancy (during the first and the third trimester, respectively) while the remaining 95% of patients (38 women) were diagnosed during post-partum and breastfeeding. In half of the cases (52.5%), the initial suspicion arose from self-palpation of a breast node, and in 85% of cases PABC was diagnosed in clinical T stage T1-2. Regarding surgical treatment, patients mainly had breast-conservative surgery (55%). Most cases were invasive ductal carcinomas (*n* = 34; 85%), four were in situ carcinomas (DCIS) and two were invasive lobular carcinomas. PABC were mainly unifocal (in 62.5% of cases) of high histologic grade (G3 in 75% of cases). LVSI and nodal involvement occurred in 45% of cases. The median follow-up was 93 months. Regarding adjuvant treatment, 33 patients (82.5%) received chemotherapy, 3 (7.5%) received trastuzumab in association, 26 (65%) received radiotherapy, and 30 (75%) received hormonotherapy (mainly tamoxifen).

### 3.2. Comparison of PABC with the Other Groups

As shown in Table 1, women with PABC or with BC diagnosed 2–5 years after pregnancy were, on average, 2.8 years younger at the time of surgery (mean age was 36 years old for both groups) than patients with BC > 10 years after pregnancy (mean age 39 years old; *p*-value = 0.014 and 0.011, respectively). No significant differences were found regarding age at diagnosis between PABC patients and nulliparous women.

Regarding histology, infiltrating ductal carcinoma represented the most frequent histotype (80–85%) in the whole population, whereas DCIS represented 10% of PABC and less than 3% in >10 years BC patients. An almost double incidence of infiltrating lobular carcinoma was found in young nulliparous women and in women diagnosed 2–5 and 5–10 years after pregnancy (9.3%, 12.5%, and 10.2%, respectively) compared with PABC (5%).

Regarding other histological features such as tumor size, node involvement, and histological grade, no significant differences were found among groups.

Negativity for ER was found in 30% of PABC, in 20.3% of nulliparous young women, and 18% of women diagnosed with BC > 2 years since pregnancy.

When the surrogate molecular classification was considered, the percentage of HER2 positive BC was significantly higher in PABC patients (10%) compared with nulliparous young women (1%) or in the >2 years since the last pregnancy group (2.2%) (*p* = 0.01).

PABC patients underwent a mastectomy in 45% of cases. Similar mastectomy rates were observed in patients diagnosed 2–5 years (45%) or 5–10 years after pregnancy (55%). Patients diagnosed with BC within 10 years after pregnancy underwent mastectomy more frequently than nulliparous patients (33.3%) and those diagnosed beyond 10 years after delivery (22.9%; *p* = 0.019), as shown in Table 2.

We have not performed analysis according to the genetic findings, due to the low sample size. Some patients in our dataset were tested for genetic predisposition (BRCA 1–2 mutation) according to international guidelines. BRCA results are available for 29 patients. Twenty-one were BRCA negative, and eight were BRCA positive. Among PABC patients only two were tested, finding negative BRCA results.

### 3.3. DFS Analysis

No differences in DFS were observed among nulliparous women, PABC patients, and women who had BC more than 2 years since the last pregnancy. Local DFS was not affected by tumor size (*p* = 0.22), histologic grade (*p* = 0.41), lymphatic (*p* = 0.44) or vascular invasion (*p* = 0.30), hormone receptor status (*p* = 0.41) and time since last pregnancy (*p* = 0.85).

**Figure 2:**
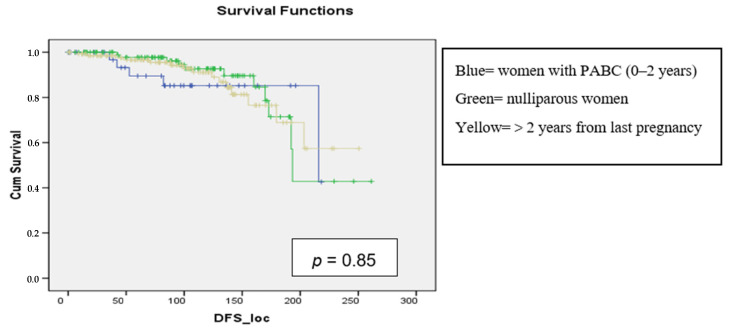
Local DFS in months according to nulliparity, multiparity, and time since last pregnancy in the analysis of 3 groups.

As shown in Figure 2, local DFS in women with PABC (in blue) showed an initial decrease, that stabilized after about 5 years from surgery. In nulliparous women, the decrease in free-from-recurrence patients was more gradual but continuous, as in women who had pregnancy more than 2 years after surgery.

**Figure 3:**
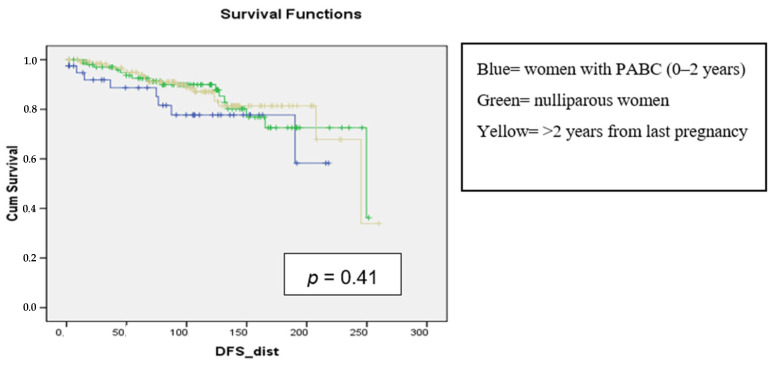
Distant DFS in month based on nulliparity, pluriparity, and time since last pregnancy in the analysis of 3 groups.

Regarding distant DFS, it was not affected by tumor size (*p* = 0.15), hormone receptor status (*p* = 0.34), and time since last pregnancy (*p* = 0.41), and patients with no distant recurrence decreased similarly for both PABC (in blue), BC patients diagnosed beyond 2 years since last pregnancy (in yellow), and young nulliparous women with BC (in green) (Figure 3).

**Figure 4:**
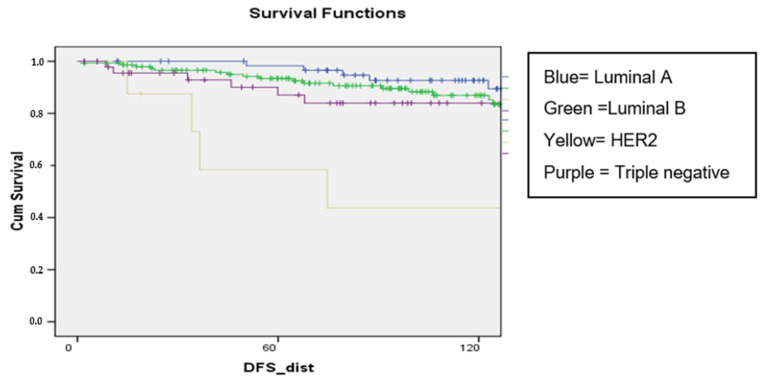
Distant DFS by subtype of the surrogate molecular classification.

In our study, distant DFS was negatively affected by lymph node involvement (*p* < 0.001), high histological grade (*p* = 0.001), presence of LVSI (*p* = 0.007 and 0.005), and by the HER2 subtype (*p* = 0.004) (Figure 4).

Univariate analysis (Table 3) showed that an increased risk for distant recurrence was affected by lymph node involvement (OR = 2.4; *p* < 0.0001), large size (T stage > 2; OR = 1.5; *p* = 0.05), high grade (OR = 2.6; *p* = 0.01), or HER2 molecular subtype (OR = 5.2; *p* = 0.01). Pregnancy and the time since delivery did not affect the risk of recurrence (OR = 0.8; *p* = 0.30).

According to multivariate analysis, lymph node involvement increased the risk of recurrence by 2.4 times independently of all other variables (*p* = 0.0001), whereas PABC and time since pregnancy were not independent negative prognostic factors for DFS (*p* = 0.824).

## 4. Discussion

BC in young women is known to be more aggressive than in older patients, associated with shorter survival and presenting more frequent genetic mutations in TP53 and BRCA1 [28]. In this context, PABC represents a specific subtype of BC occurring in young women, showing its own clinical and pathological features. In fact, during pregnancy and lactation, the breast undergoes remodeling programs that might involve inflammation pathways and may be associated with tumor development and progression. In this setting, genomic signature also varies [29], with PABC showing increased expression of immune response mediators [30]. Paris et al. [31] described three different possible mechanisms underlying PABC development: the hormonal changes that occur during pregnancy and lead to breast cell proliferation, the immunological changes that allow the establishment of an immunologic tolerance [32], and the breast tissue involutions that occur after delivery and contributes to creating an inflammatory microenvironment.

In our study, PABC was defined as BC diagnosed during pregnancy and in the first 2 years after pregnancy, following other authors [6,22]. PABC is defined through the literature in different ways; some authors consider only BC occurring during pregnancy [33], while others consider all BC diagnosed during pregnancy and lactation or up to 5 years post-partum [8]. We decided to use the definition by Johansson ALV et al. and by Gooch et al., because it allowed us to include in this group almost all women who were pregnant or were breastfeeding at the time of diagnosis (as the WHO suggests: exclusive breastfeeding in the first 6 months adequate and safe complementary foods, while continuing to breastfeed for up to two years or beyond [34]). Pregnancy and lactation are known to be a high-risk period, as the mammary gland undergoes several changes and remodeling, but further subdivision and analysis of the two groups was not possible due to the low sample size.

In our results, the mean age at surgery reflects the long-term protective effect of pregnancy recognized in the literature [35,36], resulting in delayed occurrence of BC in multiparous women.

In our population, mean age at BC diagnosis was 36.4 years old for PABC patients and 39.2 years old for patients with BC diagnosis > 10 years after pregnancy. Although these data reflect the national ones, there is now a tendency to search for pregnancy at an increasingly advanced age. Hence the need to analyze the various screening possibilities in asymptomatic but older pregnant patients. Classical screening methods (mammography) in asymptomatic pregnant women are not recommended routinely nowadays. Good practice would be to prescribe mammography among pre-conceptional exams in women who are looking to become pregnant after 40 years old and to investigate any breast lump or symptom reported by the patient. As emphasized by Galati et al. [1], it is important not to underestimate categorizing any galactocele or abscess/mastitis. It is recommended to perform a clinical breast exam during pregnancy as a part of screening, which should be performed during the first visit. Mammography is suggested by some studies 3 months after delivery or after 6 months for high-risk patients planning to breastfeed for a longer period. However, Magnetic Resonance Imaging (MRI) for high-risk breast cancer screening is not recommended during pregnancy. Ultrasound screening for high-risk breast cancer pregnant women is also not recommended, because of the increased rate of false-positive findings, unless in symptomatic patients [37].

According to the literature, in most cases, the diagnostic pathway of PABC begins with the finding of a breast lump, in contrast to non-PABC cases, in which findings by screening mammography are more common [38]. According to the data, in our study, self-palpation was the primary diagnostic method in 50% of the cases, while in two patients, a suspicious nipple discharge was the first indication of an issue. This diagnostic delay can be attributed to the higher breast density common in young women, particularly during pregnancy and lactation, as described in the literature [39,40].

Regarding the treatment of BC diagnosed during pregnancy, it strongly depends on the time of onset. According to NCCN guidelines [41], BC treatment should be as similar as possible to that offered to non-pregnant women. Surgery is always feasible during pregnancy, as well as sentinel lymph node biopsy with technetium 99. The decision between conservative or radical surgery depends on clinical variables, but also on the need to not delay radiotherapy treatment, which is contraindicated throughout pregnancy. For this reason, mastectomy might be preferred in the first trimester. On the contrary, chemotherapy can be delivered in the adjuvant or neoadjuvant setting from the 14th week of gestation without any teratogen effect, while growth restriction and preterm deliveries have been described. Weekly taxanes should be preferred. Target therapies such as trastuzumab and hormonotherapy are contraindicated during pregnancy. Delivery should be avoided during the maternal nadir, usually 2–3 weeks after chemotherapy treatment, because of possible fetal neutropenia and perinatal infection.

In the literature, PABC was associated with higher rates of mastectomies [39,42]. This can be due to a greater aggressiveness of the carcinoma itself, or to the specific treatment strategy (i.e., to avoid radiotherapy). In our sample, patients diagnosed with BC within 10 years after pregnancy underwent mastectomy more frequently than nulliparous patients and those diagnosed beyond 10 years after delivery.

Regarding histopathological features, PABC were infiltrating ductal type in 85% of the cases, <2 cm in half of the cases, and lymph nodes were involved at diagnosis in 45% of patients. PABC was characterized by high histological grade in 75% of patients, and by the presence of LVSI in 45% of cases. These results are in line with those found in the literature, where PABC is described in most cases as an aggressive, high-grade carcinoma [35,43], characterized by negativity for hormone receptors, with reduced frequency of luminal subtypes and a higher occurrence of HER2 and triple-negative carcinomas [44], as reported by Suelmann et al. on a large Dutch cohort (20% vs. 10% for HER2 positive BC and 33.8% vs. 22% for TNBC). In our sample, the percentage of HER2 overexpressing tumors was 10.5% in women with PABC, 1% in BC nulliparous patients and 2.3% in BC patients diagnosed beyond 2 years from pregnancy, although in other studies HER2 positive PABC reached 30% [39].

The study analyzed local and distant DFS and its correlation to different prognostic factors. Lymph node involvement is a known negative prognostic factor for DFS [39] and consistent with this, the study demonstrated an influence between nodal involvement and the risk of distant recurrence in our cohort of young women, in accordance with Gnerlich et al. [45].

High histologic grade and lymphatic and vascular invasion were also confirmed as negative prognostic factors for distant DFS, in line with literature data [39,46]. Patients with HER2 overexpression and TNBC are reported through literature to have lower DFS and worse prognosis than patients with luminal BC [39]. In our series, considering IHC surrogates, HER2 positive BC, but not luminal and TNBC, showed a reduction in distant DFS at 10 years after surgery.

A meta-analysis of 41 studies and 4929 cases, showed a worse DFS for women with PABC compared to non-PABC patients (HR 1.51; 95% CI 1.22–1.88) [20], and these findings were confirmed in more recent metanalysis (HR 1.39 for DFS in PABC vs. non-PABC patients) [8]. However, these meta-analyses included studies with patients who differed greatly in age, stage of disease and geographical origin and other confounding factors.

In our study, when analyzing time since pregnancy and nulliparity, there was no statistical significance for either local recurrence or distant recurrence. On the contrary, Mathelin C et al. [47] and Hatem et al. [48] found the opposite results, as PABC patients had a worse DFS and even a worse OS when considering the patients who did not receive neoadjuvant chemotherapy compared to non-pregnant age-matched BC patients.

In our study, univariate analysis confirmed the negative influence on DFS of lymph node involvement, high grade, LVSI and HER2 subtype, but not pregnancy. Multivariate analysis adjusted for confounding factors showed statistical significance only for lymph node involvement. Time from last pregnancy to BC diagnosis did not show an increased risk of recurrence, in agreement with the study by Murphy et al. [43], where at about 5 years follow-up up there were no differences in OS between PABC and non-PABC patients and on multivariate analysis, PABC was not an independent prognostic factor.

Among limitations, the relatively small sample of the PABC population and the lack of data regarding some other specific anatomopathological features (i.e., PDL-1 or TILs evaluation) must be addressed.

In addition, also due to the low sample size, we could not separately analyze women with BC diagnosed during pregnancy or breastfeeding, and not enough death events were observed to assess overall survival (OS).

Lastly, the long follow-up period is a strength of our study, but exposes the population to several biases, first and foremost the fact that over time treatment protocols have been updated and there are now other adjuvant therapies that were previously unavailable, such as the introduction of Trastuzumab in HER 2-positive tumors.

Selection bias is inherent in the characteristics of the analyzed population as it is a sample of young women with no access to screening (due to age) who were referred to our center at the arising of symptoms.

These aspects will be implemented in future research. Among research fields that could be implemented, are the impact of genetic mutation on PABC characteristics and treatment and the management of pregnancy in metastatic settings.

In addition, an unambiguous definition of PABC is needed, as well as a better differentiation of BC occurred in different moments during pregnancy or lactation to make different studies more easily comparable.

## 5. Conclusions

In this study, PABC is characterized by high histologic grade and more aggressive features such as HER2 overexpression, compared with BC diagnosed in nulliparous young women or more than two years after the last pregnancy. These features are all known to be negative prognostic factors. According to our results, pregnancy cannot be defined as an independent prognostic factor for disease progression as the factors that worsen treatment outcomes are mainly the unfavorable clinical and histological profile of the tumor rather than obstetrical history.

However, biological aggressiveness is not reflected in DFS. The lack of difference in DFS in our population could reflect the fact that PABC patients can eventually receive .optimal treatment, but further studies are needed to specifically analyze the molecular mechanisms underlying tumor development and response to treatment in this population.

A bigger sample size and an even longer follow-up will be implemented in the next years to further analyze the differences between cancer during pregnancy and lactation and to analyze the impact of pregnancy on OS. A multi-center study would be advisable to better analyze these aspects.

## Figures and Tables

**Table 1:** General characteristics among the 5 subgroups.

	Nulliparous (%)	PABC (%)	2–5 Years (%)	5–10 Years (%)	>10 Years (%)
Mean age at diagnosis	35 (SD 4.58)	36.4 (SD 3.17)	36.3 (SD 3.87)	37.6 (SD 3.08)	39.2 (SD 1.44)
IDC	87 (80.6)	34 (85)	32 (80)	49 (83.1)	31 (88.6)
DCIS	8 (7.4)	4 (10)	2 (5)	4 (6.8)	1 (2.9)
ILC	10 (9.3)	2 (5)	5 (12.5)	6 (10.2)	1 (2.9)
Others	3 (2.8)	0 (0)	1 (2.5)	0 (0)	2 (5.7)
In situ	8 (7.4)	4 (10)	2 (5)	4 (6.8)	1 (2.9)
T1	66 (61)	20 (50)	19 (47.5)	29 (49.2)	22 (62.8)
T2	26 (24)	13 (32.5)	17 (42.5)	25 (42.4)	11 (31.4)
T3-T4	8 (7.4)	3 (7.5)	2 (5)	1 (1.7)	1 (2.9)
N0	52 (48.1)	22 (55)	21 (52.5)	37 (62.7)	21 (60)
N1	41 (38)	9 (22.5)	14 (35)	15 (25.4)	10 (28.6)
N2	15 (13.9)	9 (22.5)	5 (12.5)	7 (11.9)	4 (11.4)
G1–G2	40 (37)	10 (25)	12 (30)	23 (39)	16 (45.7)
G3	68 (63)	30 (75)	28 (70)	36 (61)	19 (54.3)
Luminal A	27 (25)	5 (13.2)	10 (25)	13 (22)	10 (28.6)
Luminal B	59 (54.6)	21 (55.3)	20 (50)	36 (61)	21 (60)
HER2	1 (0.9)	4 (10)	2 (5)	1 (1.7)	0 (0.0)
TNBC	21 (19.4)	8 (20)	8 (20)	9 (15.3)	4 (11.4)

IDC: invasive ductal carcinoma, DCIS: ductal carcinoma in situ, ILC: invasive lobular carcinoma, T: clinical size according to TNM 2017, N: nodal involvement, G: histological grade, TNBC: triple-negative breast cancer.

**Table 2:** Type of surgery in the 5 subgroup populations.

Type of Surgery	Nulliparous (%)	PABC (%)	2–5 Years (%)	5–10 Years (%)	>10 Years (%)
BCS	72 (66.7)	22 (55)	22 (55)	27 (45.8)	27 (77.1)
Mastectomy	36 (33.3)	18 (45)	18 (45)	8 (54.2)	8 (22.9)

BCS: breast-conserving surgery.

**Table 3:** Univariate analysis distant DFS.

Variables	Odds Ratio	IC 95%		*p*
T Stage > 2	1.5	1	1.3	0.05
Node involvement	2.4	1.6	3.5	0.0001
High grade	2.6	1.2	5.8	0.01
Lymphatic invasion	2.3	1.2	4.3	0.009
Vascular invasion	2.3	1.2	4.5	0.007
ER positivity	0.8	0.4	1.6	0.48
HER2 molecular subtype	5.2	1.4	18.8	0.01
PABC	0.8	0.5	1.2	0.30

T stage: tumor size according to TMN 2017; ER: estrogen receptor; HER2: Human Epidermal Growth Factor Receptor 2; PABC: pregnancy-associated breast cancer.

## Data Availability

The data presented in this study are available on request from the corresponding author on reasonable request. The data are not publicly available due to privacy policies.
